# A Systematic Review and Meta-Analysis Association Between Periodontitis and Age-Related Macular Degeneration: Potential for Personalized Approach

**DOI:** 10.3390/jpm15040145

**Published:** 2025-04-05

**Authors:** Sophie Boberg-Ans, Frederikke Arnold-Vangsted, Anna Bonde Scheel-Bech, Lars Christian Boberg-Ans, Andreas Arnold-Vangsted, Christian Jakobsen, Kasper Stokbro, Yousif Subhi

**Affiliations:** 1 Nord Specialtandlæger, 2880 Kongens Lyngby, Denmark; sophieboberg-ans@hotmail.com; 2 Department of Odontology, University of Copenhagen, 2200 Copenhagen, Denmark; frede111@msn.com; 3 Department of Ophthalmology, Rigshospitalet, 2600 Glostrup, Denmark; anna.bonde.scheel-bech@regionh.dk (A.B.S.-B.); andreas.arnold-vangsted@regionh.dk (A.A.-V.); 4 Department of Ophthalmology, University Hospital of Southern Denmark, 7100 Vejle, Denmark; lars.christian.boberg-ans@rsyd.dk; 5 Department of Ophthalmology, Innlandet Hospital Trust, 2406 Elverum, Norway; 6 Department of Oral and Maxillofacial Surgery, Odense University Hospital, 5200 Odense, Denmark; christian.jakobsen@rsyd.dk (C.J.); kasper.stokbro@rsyd.dk (K.S.); 7 Department of Clinical Research, University of Southern Denmark, 5200 Odense, Denmark; 8 Department of Clinical Medicine, University of Copenhagen, 2200 Copenhagen, Denmark

**Keywords:** periodontitis, age-related macular degeneration, inflammation, association, meta-analysis

## Abstract

**Background/Objectives**: Periodontitis is a chronic inflammatory disease that leads to systemic low-grade inflammation. Systemic low-grade inflammation has been found in patients with age-related macular degeneration (AMD). In this systematic review and meta-analysis, we evaluated the association between periodontitis and AMD. **Methods**: We searched 11 scientific literature databases on 16th December 2024 for studies of a diagnosis of periodontitis and prevalent or incident AMD. Eligible studies underwent a qualitative review and meta-analysis of the association. Study selection, data extraction, and risk of bias within studies were made in duplicate by two authors and conferred with a senior author. **Results**: Seven studies eligible for review included in total 149,217 individuals. Across the seven studies, different study designs, diagnoses and definitions of periodontitis, and diagnosis and definitions of AMD were employed. Our meta-analysis showed an association between periodontitis and AMD with an odds ratio of 1.42 (95% CI: 1.12 to 1.78; *p* = 0.003). **Conclusions**: Periodontitis is significantly associated with AMD. Unlike genetic predisposition and high age, which are important risk factors of AMD that cannot be modified, periodontitis is a risk factor that can be treated and potentially eliminated, thus allowing for a personalized approach for risk elimination in AMD. Attention should be given to the dental health of patients at risk of AMD.

## 1. Introduction

Periodontitis is a chronic inflammatory disease of the gum that leads to alveolar bone loss, which can then develop into dental loss [1]. Numerous studies have linked periodontitis to systemic low-grade inflammation [1,2,3] and associated the presence of periodontitis with prevalent or incident systemic diseases, including metabolic syndrome, diabetes mellitus, and cardiovascular diseases [3]. The relationship may be multifactorial; however, a key contributor is considered to be the local gingival production of pro-inflammatory mediators of inflammation as a response to chronic bacterial infection [2,3]. Systemic leakage of pro-inflammatory molecules, such as interleukin-1, interleukin-6, and tumor necrosis factor-alpha, are all shown to contribute to systemic low-grade inflammation, as reflected by increased systemic C-reactive protein, which has been studied intensively as a harbinger of cardiovascular diseases [4,5,6,7,8].

Age-related macular degeneration (AMD) is the most prevalent cause of irreversible vision loss among the elderly in the developed world [9]. Early stages of the disease are characterized by the accumulation of lipoproteins within the Bruch’s membrane and in the sub-retinal pigment epithelium (RPE) space [10,11]. The disease can develop into a symptomatic late stage, which is characterized by either of two main features of late AMD: (1) neovascularization from the underlying choroid and into the retina, or (2) geographically demarcated areas of photoreceptor and RPE atrophy [10,11]. The etiology of AMD is considered to be a complex interplay between genetic predisposition [12,13], age-related changes of the retina and the immune system [14,15,16], and the presence of low-grade inflammation [17,18,19,20,21]. Patients with AMD have higher systemic levels of C-reactive protein and other pro-inflammatory mediators of inflammation [17,18]. The presence of low-grade inflammation is shown to increase the risk of development of AMD [21]. Evidence even suggests that an increased activity in low-grade inflammation coincides with the time of neovascular onset/progression of AMD [22]. Taken together, it would be reasonable to assume a certain pathophysiological and epidemiological relationship between the presence of periodontitis and the development of AMD, and indeed, this hypothesis has been explored in several studies. Unlike other important but unmodifiable risk factors such as genetic predisposition and high age, periodontitis is potentially a risk factor that can be treated and potentially eliminated, thus allowing for a personalized approach for risk elimination in AMD.

In this systematic review and meta-analysis, our aim was to summarize the current literature in the field and calculate a summary estimate on the association with a meta-analysis.

## 2. Materials and Methods

### 2.1. Study Design

This study was a systematic review of the literature on the association between periodontitis and AMD with a meta-analysis on the estimates of association reported across studies. Protocol preparation and methodological approach were in concordance with the recommendations of the Cochrane Handbook [23]. According to Danish law, systematic reviews do not require institutional review board approval. Our protocol was registered in the PROSPERO database (jr. no. CRD42025643558). All aspects of this study were disseminated according to the recommendations of the Preferred Reporting Items for Systematic Reviews and Meta-Analyses (PRISMA) [24].

### 2.2. Eligibility Criteria of Studies for Review

Eligible studies were defined as those who fulfilled the following criteria:

Population: Human adults who underwent a dental examination.

Exposure: Diagnosis of periodontitis.

Outcomes: Prevalent or incident AMD.

Study design: Any prospective or retrospective observational study to evaluate the association, i.e., both cross-sectional and temporal study designs were considered eligible. However, case reports, conference abstracts, non-peer-reviewed studies, and studies without original data were not considered eligible. We did restrict studies based on journal or geography. For practical purposes, we only considered studies disseminated in the English language. Studies not fulfilling these criteria were excluded.

### 2.3. Information Sources, Literature Search, and Study Selection

We systematically searched the 11 scientific literature databases PubMed, Embase, the Cochrane Central, Web of Science Core Collection, BIOSIS Previews, Current Contents Connect, Data Citation Index, Derwent Innovations Index, KCI-Korean Journal Database, Preprint Citation Index, ProQuest^TM^ Dissertations & Theses Citation Index, and SciELO Citation Index. All searches were performed by one trained author (Y.S.) on 16 December 2024. All available articles from the inception of individual databases until the date of search were included. Details of the search phrases used in individual databases are available in Supplementary File S1. One author (Y.S.) examined all titles and abstracts to remove duplicates and obviously irrelevant records. Remaining records were retrieved in full text for eligibility review. Two authors (S.B-A. and F.A-V.) independently examined full-text studies as well as reference lists for any additional potentially eligible studies. Disagreements in study eligibility were discussed between the two authors (S.B-A. and F.A-V.) and in lack of consensus, a senior author (Y.S.) made the final decision.

### 2.4. Data Collection and Risk of Bias Within Studies

Data were extracted from each study using pre-designed data extraction forms with items on study characteristics, population characteristics, methods for examination and diagnosis of periodontitis and AMD, and study results. Since we anticipated observational association studies primarily of a cross-sectional nature, we evaluated risk of bias within studies by using relevant items from the Agency for Healthcare Research and Quality (AHRQ) checklist for Cross-Sectional Studies (Questions 1–4 and 6), which is the recommended tool for such studies [25]. All data extraction and risk of bias evaluation were made by two authors (S.B-A. and F.A-V.) independently. Disagreements in data and risk of bias were discussed between the two authors (S.B-A. and F.A-V.) and in lack of consensus, a senior author (Y.S.) made the final decision.

### 2.5. Study Outcomes, Synthesis, and Risk of Bias Across Studies

The primary outcome was the association between periodontitis and AMD. These associations were presented in the qualitative review. The heterogeneity in the outcomes of available studies required us to allow alveolar bone loss as a proxy measure of periodontitis and photoreceptor layer thinning as a proxy measure of AMD. Where possible, the odds ratio (OR) on the association was extracted or calculated from the presented data. Thus, the final summary estimate in the meta-analysis was reported in OR. For meta-analysis, we used MetaXL 5.3 (EpiGear International, Sunrise Beach, QLD, Australia) for Microsoft Excel (Microsoft, Redmont, WA, USA). The random-effects model was employed to account for potential heterogeneity across studies. Heterogeneity was evaluated using the Cochran’s Q and I^2^. A funnel plot was used to evaluate risk of bias across studies. Sensitivity analysis was performed by removing each study in analysis by turn and re-calculating the summary estimate to evaluate the magnitude of change in results and the robustness of the summary estimate. Summary estimates were presented with a 95% confidence interval (95% CI).

## 3. Results

### 3.1. Study Selection

**Figure 1:**
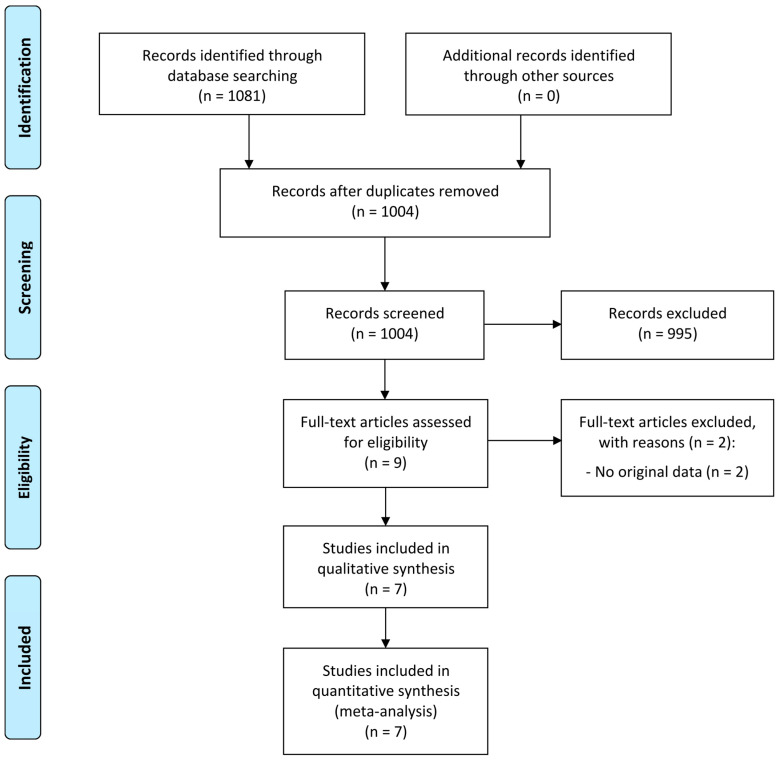
PRISMA flow diagram of the study selection process.

Our search identified a total of 1081 records, of which 77 were duplicates and 995 were obviously irrelevant. The remaining 9 records were retrieved and evaluated in full text. Of the 9 studies evaluated in full text, 2 did not present original data and were therefore not eligible according to our criteria. Thus, we included 7 studies for the qualitative and the quantitative review. The study selection process is outlined in Figure 1.

### 3.2. Study Characteristics and Populations

The seven studies eligible for review included in total 149,217 individuals [26,27,28,29,30,31,32]. Six studies were prospective, and one was retrospective in nature. Five studies were cross-sectional and two were case–control studies. Studies were conducted in the USA (*n* = 2), Finland (*n* = 1), Italy (*n* = 1), South Korea (*n* = 1), UK (*n* = 1), and Taiwan (*n* = 1). The mean age of study participants in individual studies ranged between 56 and 76 years. Details of study characteristics and participant demographics are summarized in Table 1.

Participants underwent systematic dental examination as part of the study in four studies [26,27,29,31]. In two studies, interviews and questionnaires were used to evaluate gum health and approximate a diagnosis of periodontitis [28,32]. One study used diagnosis codes from a national registry to allocate a diagnosis of periodontitis [30]. Retinal diagnosis was evaluated using fundus photography in three studies [28,29,31] and using optical coherence tomography (OCT) in two studies [26,31]. One study used diagnosis codes from a national registry to obtain knowledge of a diagnosis of AMD; however, details of the used diagnosis codes were not reported [30]. One study approximated the presence of AMD based on an interview question in which they asked patients for degenerative fundus changes [27]. Details of the diagnostic approach are summarized in Table 2.

The relationship between periodontitis and AMD was evaluated across studies while also adjusting for several factors. Studies predominantly adjusted for age, sex, and car-diovascular co-morbidities. Table 3 presents details of the variables used for adjusted analyses.

### 3.3. Results of Individual Studies

Two studies evaluated specific aspects of periodontitis and AMD [26,27]. Di Spirito et al. performed a comprehensive oral examination and performed periodontal charting with the assessment of Clinical Attachment Level, Periodontal Pocket Depth, Gingival Index, Plaque Index, Full Mouth Plaque Score, and Full Mouth Bleeding Score [26]. None of these parameters were significantly different when the authors compared 40 patients with AMD to 40 patients without AMD [26]. Karesvuo et al. examined the number of teeth, alveolar bone loss, and the presence of specific bacteria strains [27]. When comparing these parameters between individuals with AMD and individuals without AMD, the authors reported that AMD was significantly associated with a lower number of teeth and the presence of alveolar bone loss, but no specific bacteria strain [27].

Three studies evaluated the presence of a conditional association between periodontitis and AMD based on age [29,30,31]. Shin et al. reported that severe periodontal disease was only associated with an increased risk of AMD in individuals aged ≤ 62 years, not in individuals aged > 62 years [29]. Wagley et al. reported that periodontitis was only associated with an increased risk of AMD in individuals aged ≤ 60 years, not in individuals aged > 60 years [31]. The authors of both studies speculate that in older individuals, other factors may dilute the effect of periodontal disease on the risk of AMD [29,31]. Sun et al. reported that patients with periodontitis had a significantly increased risk of developing both wet and dry AMD, both in individuals aged < 65 years and in individuals aged ≥ 65 years, and that the potential risk from periodontitis increased with increasing age [30].

Two studies found evidence of an association between early retinal markers of AMD and periodontitis [28,32]. Klein et al. reported that a history of periodontal disease was associated with increased retinal pigment abnormalities, an early marker of AMD [28]. Wagner et al. investigated the association between very severe periodontitis and early markers of AMD [32]. The authors found that individuals with very severe periodontitis had significantly thinner photoreceptor layers compared to individuals without very severe periodontitis [32].

### 3.4. Risk of Bias Within Individual Studies

In our risk of bias evaluation of individual studies, we found that studies generally clearly defined the source of data and eligible criteria of the study participants. Where relevant, exclusions from analyses were explained in all cases. Apart from one study [26], time period and consecutive recruitment (or similar) were systematically also reported. Quality assurance to confirm or disregard the presence of a diagnosis of periodontitis or AMD was not performed in the majority of studies, which was identified as the main risk of bias within individual studies. Risk of bias within individual studies evaluation are presented in Table 4.

Studies are assessed on relevant items from the Agency for Healthcare Research and Quality checklist: Defines source: Defines the source of information. Eligibility criteria: Lists inclusion and exclusion criteria or refers to previous publications. Time period: Indicates the time period used for identifying participants. Consecutive recruitment: Indicates whether or not subjects were consecutively recruited for the study. Quality assurance: Describes any assessments undertaken for quality assurance purposes. Explains exclusions: Explains any patient exclusions from the analysis.

### 3.5. Meta-Analysis and Risk of Bias Across Studies

**Figure 2:**
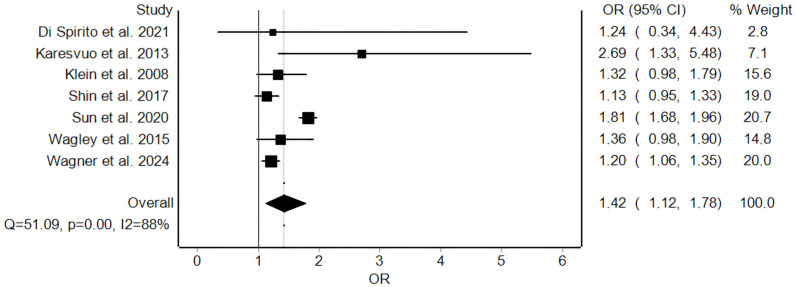
Forest plot of the meta-analysis of the association between periodontitis and age-related macular degeneration. Square dots indicate the odds ratio (OR), and whiskers indicate the 95% confidence interval (95% CI). The summary estimate is depicted with a diamond, whose length is the 95% CI. The dotted longitudinal line is the OR point of the summary estimate [26,27,28,29,30,31,32].

All studies were eligible for the quantitative analysis. Our meta-analysis showed a summary estimate of the association between periodontitis and AMD at OR 1.42 (95% CI: 1.12 to 1.78; *p* = 0.003) (Figure 2). Heterogeneity across studies was considerable at Cochran’s Q of 51.09 and I^2^ of 88%. The funnel plot was not suggestive of a risk of bias across studies (Supplementary Figure S1). Sensitivity analysis demonstrated the robustness of the summary estimate direction and amplitude, as excluding studies in turn only led to minor changes (summary estimate OR ranged within 1.23–1.49) without loss of significance (Supplementary Table S1).

## 4. Discussion

In this systematic review and meta-analysis, our aim was to summarize the literature on the association between periodontitis and AMD and to calculate a summary estimate of the association with a meta-analysis. We found that most studies provided evidence of an association using different methodological approaches, and our summary estimate is that periodontitis is associated with AMD at an OR of 1.42. Studies in this review conclude on the presence of an association after adjusting for several other confounding factors, which are independently associated with both periodontitis and AMD. Although the evidence base is limited to seven observational studies so far [26,27,28,29,30,31,32], while also considering the positive biological plausibility of a potential causal relationship, the overall picture is suggestive of a relationship that deserves further investigative efforts.

Two studies in this review evaluated the presence of AMD based on a comprehensive ophthalmic examination including OCT-based diagnosis [26,32]. In modern ophthalmic practice, OCT is considered a minimum for accurate diagnosis of AMD [33,34,35]. A diagnosis of AMD based on diagnosis codes challenges the accuracy of the diagnosis without the presence of any valid studies in the field. Diseases that mimic features of AMD without a diagnosis code of its own are at risk of being coded as AMD (e.g., central serous chorioretinopathy with macular neovascularization, dome-shaped macula, idiopathic macular neovascularization in the elderly, polypoidal choroidal vasculopathy, pattern dystrophy, and early-onset drusen spectrum diseases) [36,37,38,39,40]. In one study, the diagnosis of AMD was based on whether the participant would confirm in an interview question the presence of a doctor’s diagnosis of degenerative fundus changes [27]. Even in the presence of a reasonable validity of interview-based estimating of retinal disease, degenerative fundus changes can be due to a broad spectrum of retinal diseases, of which AMD is just one [36,37,38,39,40]. These circumstances present a certain bias around the diagnosis of AMD across studies. Similarly, the diagnosis of periodontitis was subject to different approaches with arguably different expected validity. Four studies performed dental examinations and included a variating spectrum of measures to diagnose periodontitis [26,27,29,31], of which only one study [26] followed the 2017 classification for periodontitis [41]. In the remaining three studies, one study based the presence of periodontitis on diagnosis codes [30], and two studies were based on questionnaires [28,32]. The heterogeneity of the approach to periodontitis also presents an important source of bias. However, it should also be acknowledged that large-scale studies are costly and cannot always accommodate to the best possible diagnostic practice [42]. These studies are typically designed with a consideration of cost-effectiveness in order to answer a set of research questions [42].

It is worthwhile to consider the impact of an OR of 1.42 on the association between periodontitis and AMD. A history of smoking is associated with AMD at an OR of 1.78–3.58 [43], and a history of regular physical activity is associated with AMD at an OR of 0.59–0.92 [44]. Although no interventional studies have examined smoking cessation or initiation of physical activity for patients at risk of AMD, based on the available data and the general health benefits of not smoking and being physically active, it is generally recommended that individuals at risk of AMD avoid smoking and engage in regular physical activity. This advice also possesses a biological plausibility, as it may modify the level of low-grade systemic inflammation [45]. Similarly, considering the relationship between periodontitis and AMD at an OR of 1.42, and the general health benefits of dental health and hygiene, it would be reasonable to suggest that individuals at risk of AMD who experience dental problems seek dental examination and care. Although interventional smoking cessation studies and interventional physical activity studies in the elderly can be extremely difficult, it is interesting to consider that intensive dental care as an intervention may be much more feasible. Such studies are warranted for more accurate insight into the value of intensive dental care for the risk of AMD.

The association between periodontitis and AMD has been explored meta-analytically in three previous studies [46,47,48]. These previous reviews of the evidence, summarized in years 2019–2020, all find an association between periodontitis and AMD, based on the four [47] or five studies [46,48] included in the analyses. The Cochrane Handbook recommends that systematic reviews should be routinely updated to reflect the best evidence, exemplified by 23% of reviews being out of date within two years [23]. The findings of this systematic review fall in line with the previous papers in the field but provide an updated status of the field.

Limitations of this systematic review and meta-analysis should be considered when interpreting its results. First, different study designs, diagnosis and definitions of periodontitis, and diagnosis and definitions of AMD, all challenge the accuracy of the summary estimates. Specifically, in one study, OCTs were segmented automatically, and the thickness of the RPL and RPE-BM were used as a pseudo measure of AMD rather than a routine retinal diagnosis of AMD [32]. Second, variables adjusted in the individual studies vary, which influence the potential influence of confounding factors on the estimates used in the meta-analysis. Depending on the severity of this, we may overestimate the association between periodontitis and AMD. For example, poor dietary choices and obesity are associated with both periodontitis and AMD. Finally, the strength of systematic reviews depends on the studies included, and in this study, only seven studies were available. More studies would have provided more robust conclusions; however, it should be noted that our sensitivity analyses showed robustness in the conclusion of a positive association between periodontitis and AMD.

## 5. Conclusions

Periodontitis is associated with AMD. Unlike other important but unmodifiable risk factors such as genetic predisposition and high age, periodontitis is a risk factor that can be treated and potentially eliminated, thus allowing for a personalized approach for risk elimination in AMD. Attention should be given to the dental health of patients at risk of AMD. However, more studies are warranted to understand the impact of eliminating periodontitis in patients with periodontitis and how that modulates the risk of AMD.

## Figures and Tables

**Table 1:** Characteristics of studies in review.

Reference	Country	Study Design	Population Description	Demographics
Di Spirito et al., 2021 [26]	Italy	Prospective case–control study	Cases were patients with AMD diagnosed at the local department of ophthalmology. Controls were individuals who sought routine oral examinations at the local department of dentistry. Controls were matched for age ±3 years and gender, and did not undergo eye examination. Individuals were not included in case of known advanced cataract or other ocular diseases, edentulism, oral and systemic infections, medication-related osteonecrosis of the jaws, periodontal treatment, antibiotic or corticosteroid therapy in the last 3 months.	Cases (*n* = 40) were aged mean 75.8 years. Controls (*n* = 40) were aged mean 71.2 years. Females constituted 62.5% of all participants (both cases and controls).
Karesvuo et al., 2013 [27]	Finland	Prospective cross-sectional study	A national survey study in Finland, which in a subsample, recruited 1799 individuals for oral health examination. Of these, 1751 individuals had data to determine whether or not there was AMD. Presence of AMD was based on self-reported answers to survey questions.	Individuals with AMD (*n* = 54) were aged mean 66.5 ± 13.4 years and 70% were females. Individuals without AMD (*n* = 1697) were aged mean 51.3 ± 12.7 years and 55% were females. Individuals with AMD had a higher incidence of diabetes and had a higher systolic blood pressure.
Klein et al., 2008 [28]	USA	Prospective cross-sectional study	The MESA study in 6 communities in the USA recruited 5887 individuals with gradable fundus images for the evaluation of retinal pathologies. A questionnaire was used to obtain information about past medical history, including periodontal disease.	Demographics specific for AMD or no AMD were not reported. The entire study population (*n* = 5887) had a mean age of 61.5 ± 10.0 years. Sex distribution was not reported.
Shin et al., 2017 [29]	South Korea	Prospective cross-sectional study	The KNHANES is a population-based cross-sectional survey in South Korea, which each year samples from the South Korean population for a health interview survey, nutritional survey, and health examinations, including ophthalmic and periodontal examinations. Ophthalmic examinations included fundus photography. Dental examinations were made by dentists.	Individuals with AMD (*n* = 901) were aged mean 64.8 ± 12.0 years and 62% were females. Individuals without AMD (*n* = 12,171) were aged mean 53.9 ± 22.1 years and 51% were females. Hypertension was more prevalent among individuals with AMD.
Sun et al., 2020 [30]	Taiwan	Retrospective case–control study	The Taiwan National Health Insurance Database is a compulsory system in the Taiwanese healthcare system, which registers all interactions. From this system, cases were defined as those with periodontitis, and controls were defined as those without periodontitis who were otherwise matched in terms of age and sex. Presence of periodontitis and AMD was based on diagnosis codes in the database. All were aged at least 50 years, and none had AMD at the time of index year.	A total of 83,322 individuals (41,661 with periodontitis and 41,661 without periodontitis) were followed for 15 years. Overall mean age was 60 years, and 51% were females.
Wagley et al., 2015 [31]	USA	Prospective cross-sectional study	The NHANES III is a national survey of health and nutrition in the USA, which includes evaluation of oral and retinal health. Using a mobile examination center, periodontal examinations were performed by a dentist, and retinal photographs were obtained for one randomly selected eye for retinal examination.	A total of 8208 individuals (940 with AMD and 7268 without AMD) were examined. Overall, 68% were older than 60 years, and 52% were females.
Wagner et al., 2024 [32]	UK	Prospective cross-sectional study	The UK Biobank is a cohort of more than 500,000 individuals in the UK. Participants were asked about dental problems experienced within the last year. A subset of these individuals additionally underwent a detailed ophthalmic evaluation that included retinal imaging.	A total of 36,897 individuals (1571 with severe periodontitis and 35,326 without severe periodontitis) were examined. Overall, mean age was 56 ± 8 years, and 54% were females.

Abbreviations: AMD = age-related macular degeneration; MESA = Multi-Ethnic Study of Atherosclerosis; KNHANES = Korean National Health and Nutrition Examination Survey; NHANES = National Health and Nutrition Examination Survey; UK = United Kingdom; USA = United States of America.

**Table 2:** Diagnostic details of studies.

Reference	Evaluation of Periodontitis	Evaluation of Age-Related Macular Degeneration
Di Spirito et al., 2021 [26]	Oral examination with a periodontal full-mouth charting and panoramic radiograph. Total number of teeth were recorded. Periodontal charting included assessment of CAL, PPD, GI, and PlI, all registered as six values around each tooth. Tooth mobility and class furcation were recorded. FMPS% and FMBS% were calculated. Panoramic X-rays were scored and assigned to RBL stages, and also to alveolar bone loss classes. Periodontitis case definition was performed for both cases and controls according to the 2017 classification of periodontal and peri-implant diseases and conditions.	Ophthalmic examination including BCVA, slit-lamp examination, IOP measurement, fundus examination, and SD-OCT. Ophthalmic examination was only performed on cases known to have AMD, and controls were not subject to ophthalmic examination. Cases were graded according to early AMD or late AMD with either neovascular AMD or geographic atrophy.
Karesvuo et al., 2013 [27]	Number of teeth was noted. Panoramic radiographs were taken. Alveolar bone loss was analyzed from the radiographs on mesial and distal surfaces of each tooth, in which a bone pocket was defined as a vertical deformity within the bone that extends from the alveolar bone crest apically at least to the middle third of the root. Saliva samples were analyzed for bacterial DNA.	The participants were subject to a home interview, which included the question: “Has a doctor diagnosed one of the following diseases: cataract, glaucoma, degenerative fundus changes, or other visual defect or injury?”. If the participant responded with the presence of degenerative fundus changes, this was accepted as a marker of AMD.
Klein et al., 2008 [28]	A detailed questionnaire was used to determine presence of medical comorbidities. One of these questions was: “Has a dentist ever told you that you had periodontitis or gum disease?”	All participants underwent 45-degree fundus photography centered on the fovea without pupillary dilation. Early AMD was defined by either the presence of any soft drusen or pigmentary abnormalities. Late AMD was defined by the presence of geographic atrophy, subretinal hemorrhage, visible subretinal neovascularization, or subretinal fibrous scar.
Shin et al., 2017 [29]	The World Health Organization CPI was used to assess periodontal conditions and defined periodontal disease as a CPI ≥ 3. Periodontal tissues of permanent index teeth in each area were evaluated and included in the examination of bleeding upon the application of 20 g of pressure using a CPI probe, the presence of dental plaque, and the presence of periodontal pockets with measurable depths. The CPI scored on a score of 0 to 4 based on findings. A score of 0–2 points was defined as absence of periodontal disease, 3 points was defined as mild periodontal disease, and 4 points was defined as severe periodontal disease.	All participants underwent 45-degree fundus photography centered on the fovea without pupillary dilation. Early AMD was defined as presence of drusen and/or pigment abnormalities. Late AMD was defined as the presence of neovascularization or geographic atrophy. In this study, the authors categorized their data into either any AMD (both early or late AMD) or no AMD.
Sun et al., 2020 [30]	Presence of ICD-9-CM diagnosis codes 523.3 and 523.4.	Not outlined in detail. However, the authors distinguish between nonexudative and exudative AMD.
Wagley et al., 2015 [31]	Buccal and mesial-buccal aspects of each tooth from one randomly assigned upper quadrant and one randomly assigned lower quadrant were scored for loss of attachment. Level of periodontal attachment was reported in mm and calculated by measuring the distance from the cementoenamel junction to the bottom of the sulcus. Periodontal disease was defined as >10% sites with >3 mm of loss of attachment.	All participants underwent 45-degree fundus photography centered between the optic nerve head and the macula. Photographs were obtained without pupillary dilation. Early AMD was defined as presence of drusen and/or pigment abnormalities. Late AMD was defined as the presence of geographic atrophy, subretinal hemorrhage, subretinal fibrous scar, or serous subretinal detachment. In this study, the authors categorized their data into either any AMD (both early or late AMD) or no AMD.
Wagner et al., 2024 [32]	Questionnaire-based evaluation. Individuals reporting painful gums or loose teeth were considered as having very severe periodontitis. A sensitivity analysis was made by only including those with self-reported loose teeth.	All participants underwent retinal imaging using Topcon 3D-OCT, which included 45-degree fundus photography and a 6.0 × 6.0 mm OCT scan of the macula. OCTs were segmented automatically using proprietary Topcon software, and the thickness of the RPL and RPE-BM were used as a pseudo measure of AMD.

Abbreviations: 3D-OCT = 3-dimensional optical coherence tomography; AMD = age-related macular degeneration; BCVA = best-corrected visual acuity; CAL = Clinical Attachment Level; CPI = Community Periodontal Index; DNA = deoxyribonucleic acid; FMBS% = Full Mouth Bleeding Score; FMPS% = Full Mouth Plaque Score; GI = Gingival Index; mm = millimeters; ICD = International Classification of Diseases; OCT = optical coherence tomography; PlI = Plaque Index; PPD = Periodontal Pocket Depth; RBL = Radiographic Bone Loss; RPE-BM = the retinal pigment epithelium-Bruch’s membrane complex; RPL = retinal photoreceptor layer; SD-OCT = spectral domain optical coherence tomography.

**Table 3:** Variables adjusted for the association between periodontitis and AMD in the individual studies.

Reference	Variables Adjusted
Di Spirito et al., 2021 [26]	No adjustment for co-variates in the analyses, but the study attempted to recruit cases and controls that were homogenous in relation to BMI, blood pressure, hypertension, and total cholesterol (methodologically unclear).
Karesvuo et al., 2013 [27]	Age, diabetic status, smoking, systolic blood pressure, and the carriage of salivary bacteria.
Klein et al., 2008 [28]	Age, sex, race/ethnicity, and study site.
Shin et al., 2017 [29]	Age, sex, education level, household income, smoking, hypertension, CVD, anemia, hepatitis B infection, serum HDL level, BMI, serum ferritin level, and WBC count.
Sun et al., 2020 [30]	Age, sex, hypertension, diabetes, hyperlipidemia, asthma/COPD, CLD, and CKD.
Wagley et al., 2015 [31]	Age, sex, race, education, PIR, smoking status, BMI, hypertension, CVD, and CRP
Wagner et al., 2024 [32]	Age, sex, ethnicity, socioeconomic status, diabetes, hypertension, alcohol drinker status, smoking status, refractive error, and previous cataract surgery.

Abbreviations: BMI = body mass index; CKD = chronic kidney disease; CLD = chronic liver disease and cirrhosis; COPD = chronic obstructive pulmonary disease; CRP = C-reactive protein; CVD = cardiovascular disease; HDL = high-density lipoprotein; PIR = poverty income ratio; WBC = white blood cell count.

**Table 4:** Risk of bias within individual studies.

Reference	Defines Source	Eligibility Criteria	Time Period	Consecutive Recruitment	Quality Assurance	Explains Exclusions
Di Spirito et al., 2021 [26]	Yes	Yes	No	No	No	Not relevant
Karesvuo et al., 2013 [27]	Yes	Yes	Yes	Yes	No	Yes
Klein et al., 2008 [28]	Yes	Yes	Yes	Yes	No	Yes
Shin et al., 2017 [29]	Yes	Yes	Yes	Yes	Yes	Yes
Sun et al., 2020 [30]	Yes	Yes	Yes	Yes	No	Not relevant
Wagley et al., 2015 [31]	Yes	Yes	Yes	Yes	Yes	Yes
Wagner et al., 2024 [32]	Yes	Yes	Yes	Yes	Yes	Yes

## Data Availability

The original contributions presented in this study are included in the article/Supplementary Materials. Further inquiries can be directed to the corresponding author.

## Supplementary Materials

The following Supporting Information can be downloaded at: https://www.mdpi.com/article/10.3390/jpm15040145/s1, Supplementary File S1: Documentation of the literature search; Supplementary Figure S1: Funnel plot for the evaluation of risk of bias across studies; Supplementary Table S1: Sensitivity analysis of the summary estimate.
