# Supplementary material for: A Systematic Review and Meta-Analysis Association Between Periodontitis and Age-Related Macular Degeneration: Potential for Personalized Approach
Source: J Pers Med. 2025 Apr 5;15(4):145. doi: 10.3390/jpm15040145 (PMC12028726; doi:10.3390/jpm15040145)

Supplementary File S1. Documentation of the literature search.

PubMed

History and Search Details

Download Delete

Search	Actions	Details	Query	Results	Time
#1	•••	⌄	Search: **("macular degeneration" OR AMD OR ARMD) AND (periodontitis OR periodontal)** Sort by: **Most Recent**<br>("macular degeneration"[All Fields] OR ("arch med deporte"[Journal] OR "amd"[All Fields]) OR "ARMD"[All Fields]) AND ("periodontal"[All Fields] OR "periodontally"[All Fields] OR "periodontically"[All Fields] OR "periodontics"[MeSH Terms] OR "periodontics"[All Fields] OR "periodontic"[All Fields] OR "periodontitis"[MeSH Terms] OR "periodontitis"[All Fields] OR "periodontitides"[All Fields] OR ("periodontal"[All Fields] OR "periodontally"[All Fields] OR "periodontically"[All Fields] OR "periodontics"[MeSH Terms] OR "periodontics"[All Fields] OR "periodontic"[All Fields] OR "periodontitis"[MeSH Terms] OR "periodontitis"[All Fields] OR "periodontitides"[All Fields]))<br>**Translations**<br>**AMD:** "Arch Med Deporte"[Journal:__jid9112189] OR "amd"[All Fields]<br>**periodontitis:** "periodontal"[All Fields] OR "periodontally"[All Fields] OR "periodontically"[All Fields] OR "periodontics"[MeSH Terms] OR "periodontics"[All Fields] OR "periodontic"[All Fields] OR "periodontitis"[MeSH Terms] OR "periodontitis"[All Fields] OR "periodontitides"[All Fields]<br>**periodontal:** "periodontal"[All Fields] OR "periodontally"[All Fields] OR "periodontically"[All Fields] OR "periodontics"[MeSH Terms] OR "periodontics"[All Fields] OR "periodontic"[All Fields] OR "periodontitis"[MeSH Terms] OR "periodontitis"[All Fields] OR "periodontitides"[All Fields]	47	16:40:37

Showing 1 to 1 of 1 entries

EMBASE

Embase <1974 to 2024 December 13>

1 "macular degeneration".mp. [mp=title, abstract, heading word, drug trade name, original title, device manufacturer, drug manufacturer, device trade name, keyword heading word, floating subheading word, candidate term word] 47211
2 macular degeneration/ 6422
3 AMD.mp. [mp=title, abstract, heading word, drug trade name, original title, device manufacturer, drug manufacturer, device trade name, keyword heading word, floating subheading word, candidate term word] 28863
4 ARMD.mp. [mp=title, abstract, heading word, drug trade name, original title, device manufacturer, drug manufacturer, device trade name, keyword heading word, floating subheading word, candidate term word] 1375
5 periodontitis.mp. [mp=title, abstract, heading word, drug trade name, original title, device manufacturer, drug manufacturer, device trade name, keyword heading word, floating subheading word, candidate term word] 53163
6 periodontitis/ 35607
7 periodontal.mp. [mp=title, abstract, heading word, drug trade name, original title, device manufacturer, drug manufacturer, device trade name, keyword heading word, floating subheading word, candidate term word] 104923
8 1 or 2 or 3 or 4 56479
9 5 or 6 or 7 124744
10 8 and 9 91

Cochrane Library

Search Name:
Date Run: 16/12/2024 18:48:00
Comment:

ID	Search	Hits
#1	"macular degeneration" OR AMD OR ARMD	4894
#2	periodontitis OR periodontal	16543
#3	#1 AND #2	14

Web of Science Core Collection, BIOSIS Citation Index, BIOSIS Previews, Current Contents Connect, Data Citation Index, Derwent Innovations Index, KCI-Korean Journal Database, ProQuest™ Dissertations & Theses Citation Index, and SciELO Citation Index

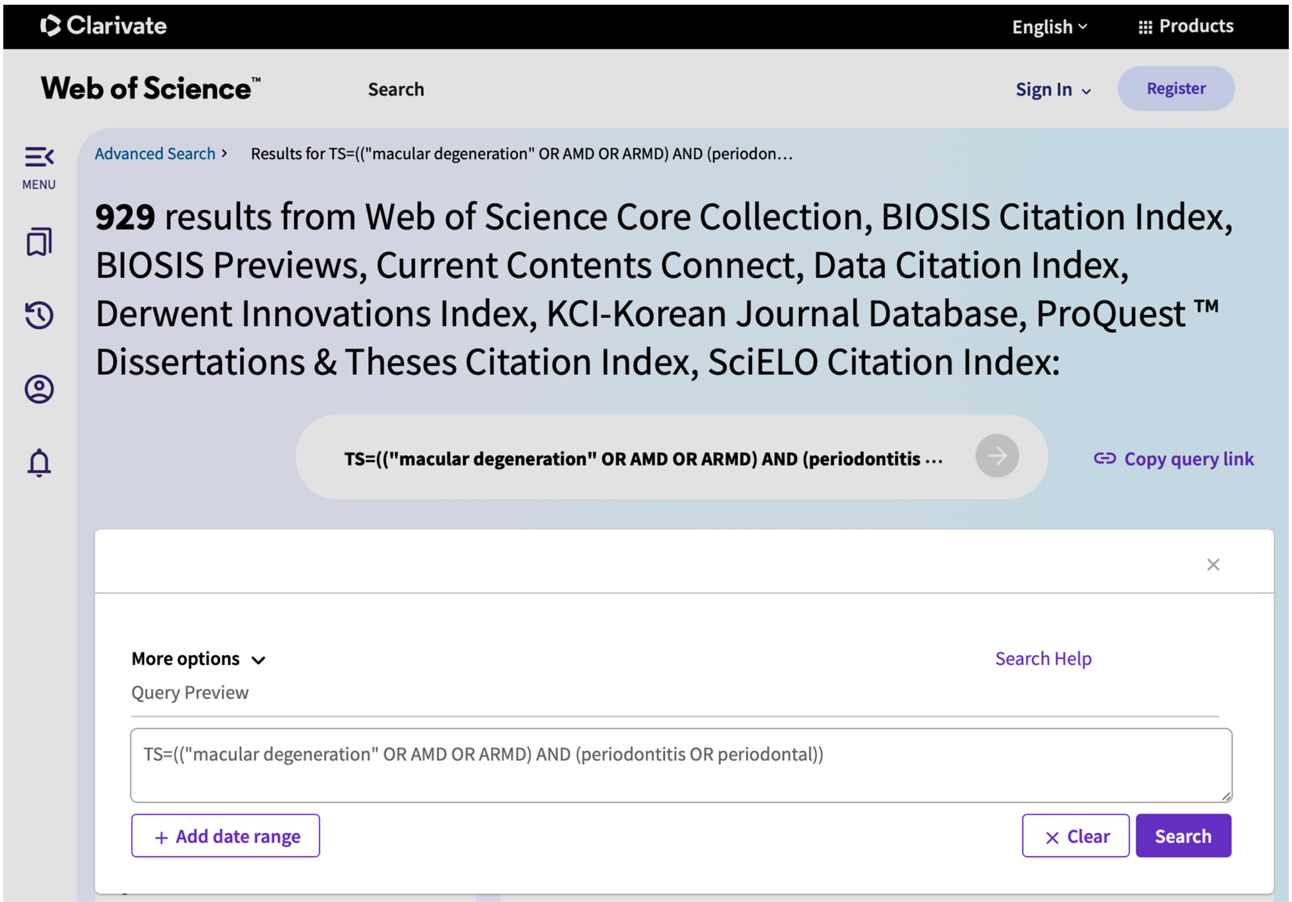

Supplement: Supplementary file 1 [file jpm-15-00145-s001.zip › Supplementary File S1.pdf]
