# Supplementary material for: A Systematic Review and Meta-Analysis Association Between Periodontitis and Age-Related Macular Degeneration: Potential for Personalized Approach
Source: J Pers Med. 2025 Apr 5;15(4):145. doi: 10.3390/jpm15040145 (PMC12028726; doi:10.3390/jpm15040145)
Supplement: Supplementary file 1 [file jpm-15-00145-s001.zip › Supplementary Table S1.pdf]

Supplementary Table S1. Sensitivity analysis of the summary estimate.

Excluded study	Pooled OR	LCI 95%	HCI 95%	Cochran's Q	I2
Di Spirito et al. 2021	1.42	1.12	1.80	50.98	90.19
Karesvuo et al. 2013	1.35	1.06	1.71	48.59	89.71
Klein et al. 2008	1.44	1.11	1.87	50.18	90.04
Shin et al. 2017	1.49	1.16	1.92	37.28	86.59
Sun et al. 2020	1.23	1.10	1.39	6.35	21.25
Wagley et al. 2015	1.43	1.10	1.85	50.62	90.12
Wagner et al. 2024	1.48	1.13	1.93	30.67	83.70
