# Supplementary figures and images for: A Systematic Review and Meta-Analysis Association Between Periodontitis and Age-Related Macular Degeneration: Potential for Personalized Approach
Source: J Pers Med. 2025 Apr 5;15(4):145. doi: 10.3390/jpm15040145 (PMC12028726; doi:10.3390/jpm15040145)

**Supplementary Figure S1.** Funnel plot for the evaluation of risk of bias across studies.

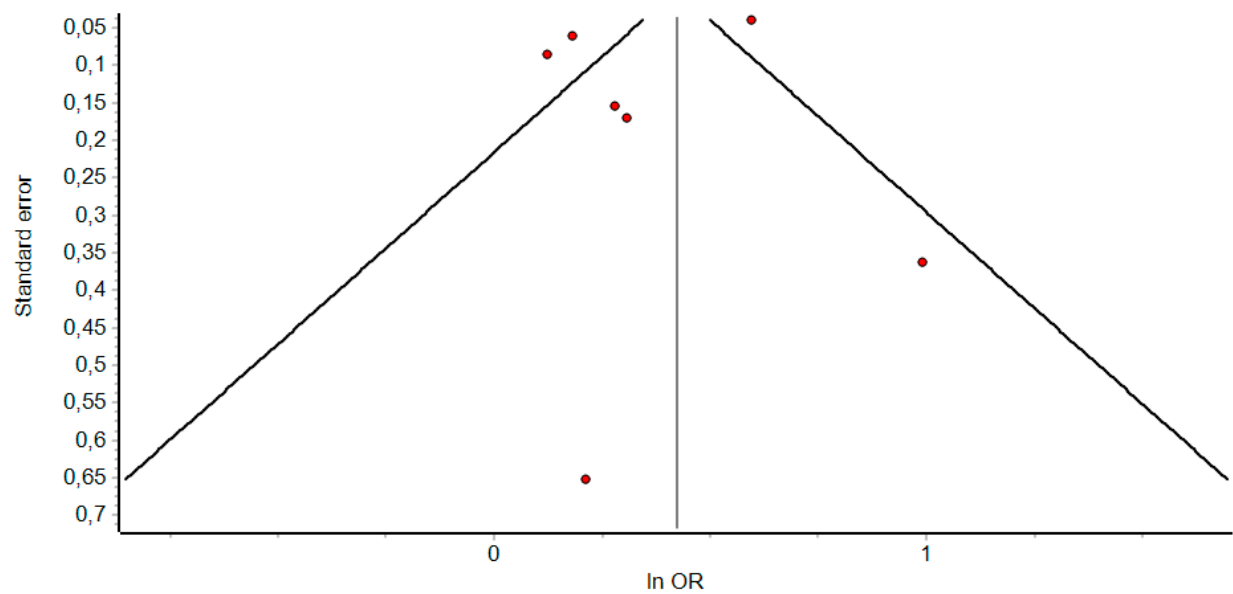

Supplement: Supplementary file 1 [file jpm-15-00145-s001.zip › Supplementary Figure S1.pdf]
